# Transosseous Suture Fixation of Avulsion Fracture of the Ulnar Collateral Ligament of the Thumb Metacarpophalangeal Joint

**DOI:** 10.7759/cureus.7902

**Published:** 2020-04-30

**Authors:** Panagiotis V Samelis

**Affiliations:** 1 First Orthopaedic Department, Children’s General Hospital Panagiotis & Aglaia Kyriakou, Athens, GRC; 2 Orthopaedics, Orthopaedic Research and Education Center, Attikon University Hospital, Athens, GRC

**Keywords:** ucl, ulnar, collateral, ligament, thumb, avulsion, fracture, transosseous, fixation, stener

## Abstract

Acute traumatic avulsion or rupture of the ulnar collateral ligament (UCL) of the thumb metacarpophalangeal (TMP) joint is a frequent sports-related injury. If not diagnosed and treated early, it may lead to chronic instability, pain, and loss of pinch and grip strength and possibly osteoarthritis. UCL insufficiency may be treated by various techniques, such as bone anchors, transosseous sutures with or without a pull-out button, tendon grafts, or with TMP joint fusion in neglected cases with arthrosis. A simple technique of internal fixation of an avulsion fracture of the distal attachment of the UCL on the proximal phalanx of the thumb using transosseous sutures is described.

## Introduction

Described as early as 1939, acute ulnar collateral ligament (UCL) insufficiency was originally considered a wintersports-related injury of the thumb metacarpophalangeal joint (TMP), and hence the synonym “skier’s thumb” [1]. The term “gamekeeper’s thumb” has been coined some years later, not as a sports-related acute injury, but as the result of repetitive trauma when gamekeepers tried to kill a rabbit by twisting its neck [2]. Loss of TMP joint stability is a serious obstacle to normal pinch and grip, and it imposes significant risk for future osteoarthritis of the joint [3,4].

The incidence of UCL insufficiency in the USA is estimated at 200,000 patients per year. It is the most frequent injury of the base of the thumb (86%) [3,4]. It is usually the result of an acute valgus stress on the TMP joint after a fall on the outstretched hand with the thumb in abduction. Acute pain, swelling, and bruising around the thenar follow. Local tenderness is elicited. The ligament may be stretched but intact (grade I), partially ruptured (grade II), or completely ruptured (grade III). Complete ruptures almost always affect the distal end of the ligament [4,5]. In 25% of complete UCL ruptures, an avulsion fracture is evident at the base of the proximal phalanx of the thumb (25%) [1,4]. The majority (up to 87%) of complete ruptures of UCL present a Stener lesion, that is, interposition of the adductor pollicis aponeurosis between the injured ends of the ligament [6]. A Stener lesion is an absolute indication for surgical treatment [6]. History, clinical examination, and routine hand x-rays usually suffice to make the diagnosis of an unstable TMP joint. A 30-degree overall valgus laxity of the TMP joint or a 15-degree difference from the contralateral TMP joint is generally thought to confirm diagnosis of a complete UCL injury [5]. Stress testing after lidocaine injection and MRI of the injured joint have also been implemented in the diagnosis of a complete UCL rupture [3].

Surgical treatment within four weeks after injury is recommended in dislocated or unstable injuries [3]. A fragment is considered to be dislocated if it is displaced more than 1 mm or if it is malrotated [4]. Lack of a firm endpoint on a valgus stress indicates an unstable UCL rupture [3,7].

The aim of this report is to describe an easily applied, low-cost surgical technique for the anatomic fixation of the distally avulsed bony attachment of UCL, using transosseous sutures.

## Case presentation

**Figure 1:**
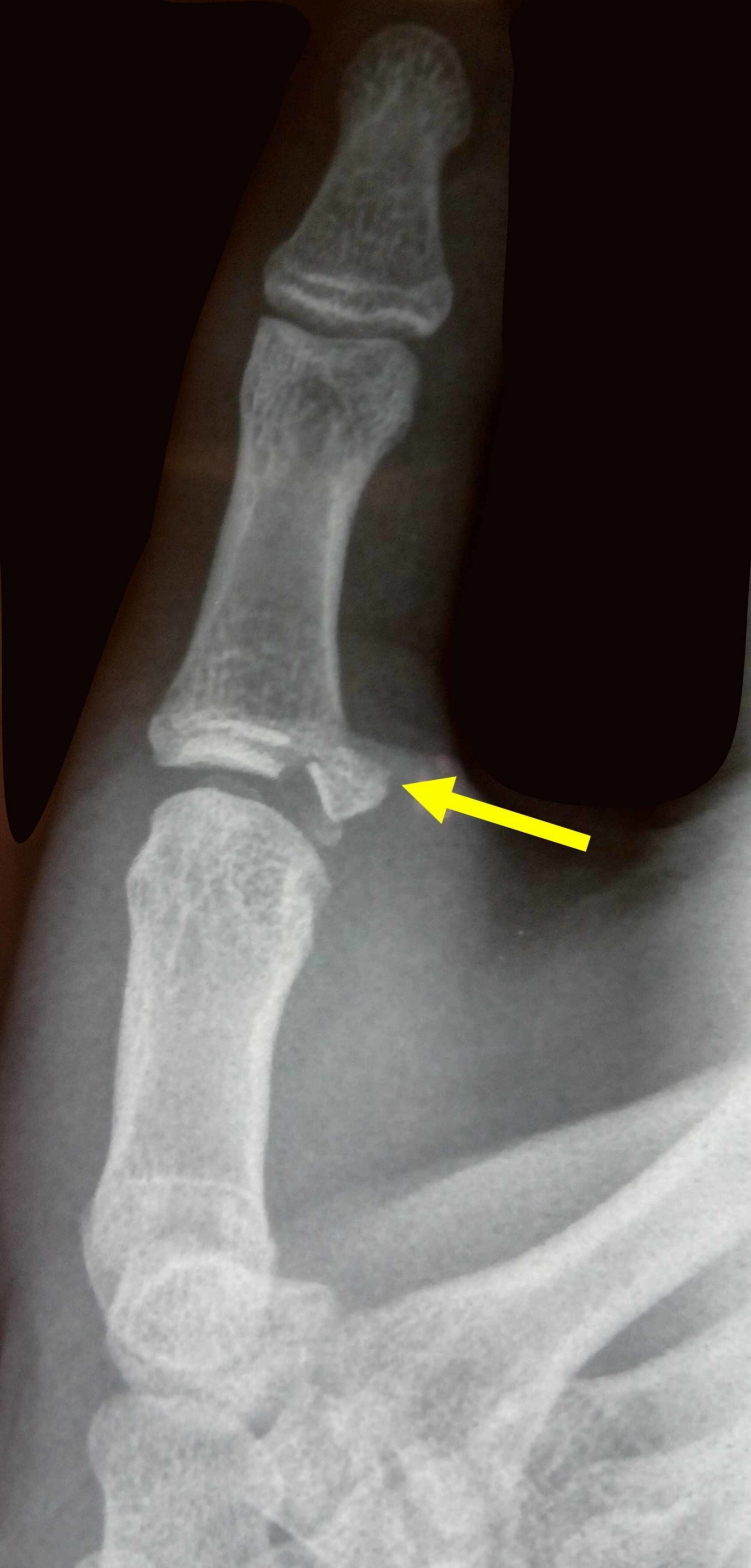
X-ray view of the right thumb of a 15-year-old boy after a fall from his bicycle The yellow arrow indicates the avulsion fracture of the attachment of the ulnar collateral ligament of the thumb metacarpophalangeal joint.

A 15-year-old boy presented at the emergency department with a painful, swollen hand after a fall from his bicycle. On x-rays, a displaced avulsion fracture at the distal attachment of the UCL was diagnosed. The fracture was displaced more than 1 mm and malrotated. The size of the avulsed fracture was more than one-third of the joint surface (Figure 1).

**Figure 2:**
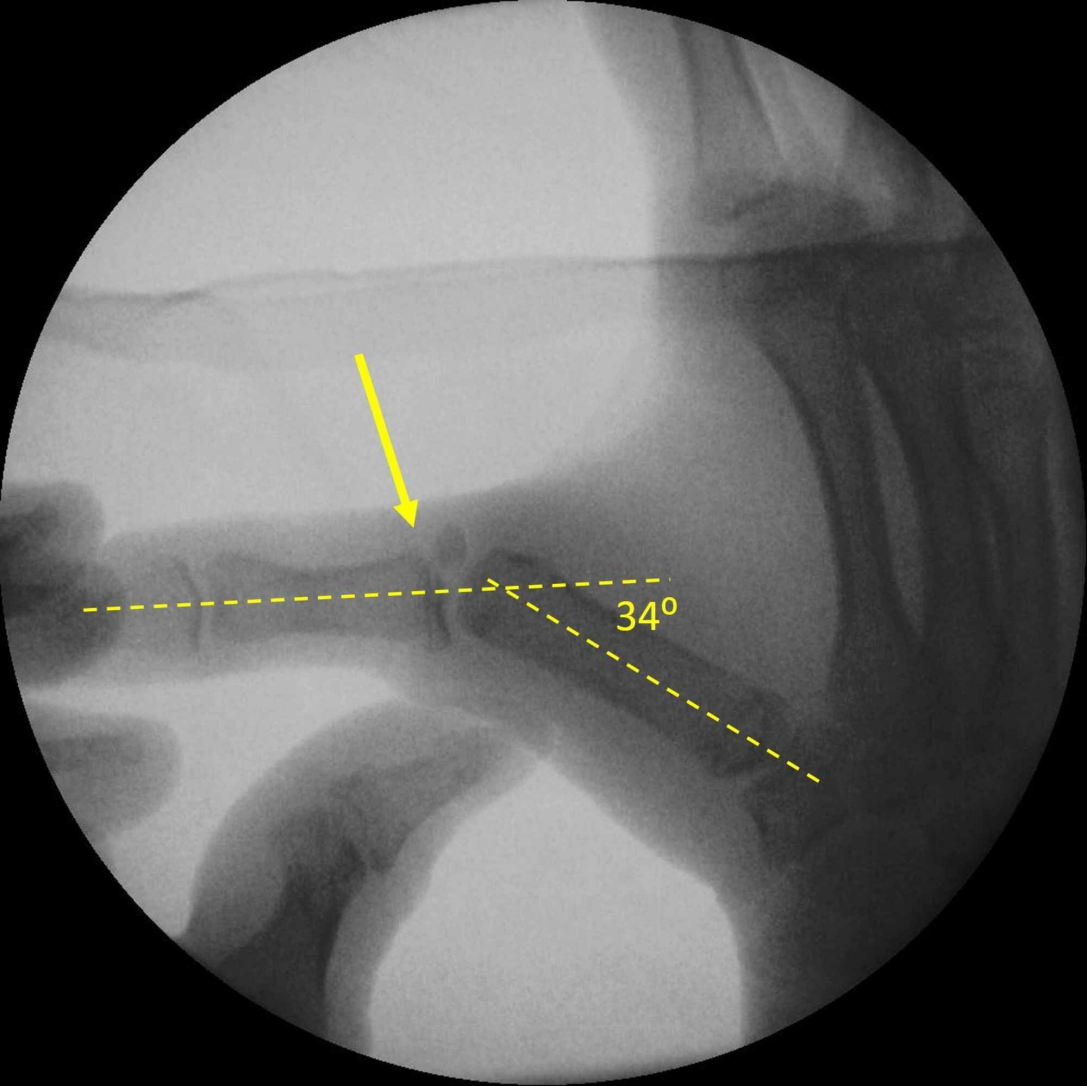
Valgus stress test of the TMP joint under general anesthesia Valgus laxity of 34 degrees at the TMP joint and further displacement of the avulsed fracture fragment are evident (yellow arrow). TMP: thumb metacarpophalangeal joint.

Surgical treatment was decided. Instability was confirmed under general anesthesia. The TMP joint angulated 34 degrees when a valgus stress was applied. Further displacement of the fracture was evident with valgus stress (Figure 2).

**Figure 3:**
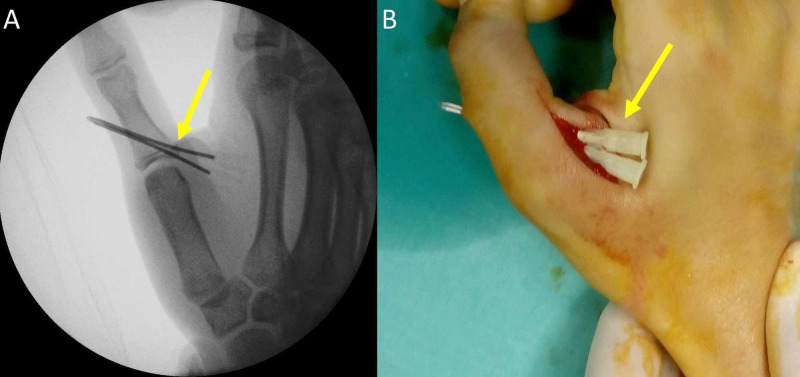
Needle insertion through the bony avulsion of the UCL and the proximal phalanx of the thumb (A) Image intensification. (B) Intraoperative view. UCL: ulnar collateral ligament

A curved dorsomedial skin incision over the TMP joint was performed. The dorsal cutaneous branch of the radial nerve was identified and protected. The aponeurosis of the adductor pollicis muscle was cut transversely, and the UCL was inspected. The fracture gap at the base of the proximal phalanx of the thumb was cleared of debris and clots. The avulsed bony attachment of the UCL was reduced. Under image intensification and gentle oscillating movements (free-hand), two 16-gauge needles were inserted through this fragment. The needles were further advanced into the proximal phalanx of the thumb. They converged and penetrated the radial cortex of the proximal phalanx and the skin. A very small (1-2 mm) incision was made to connect the exit points of the two needles (Figure 3).

**Figure 4:**
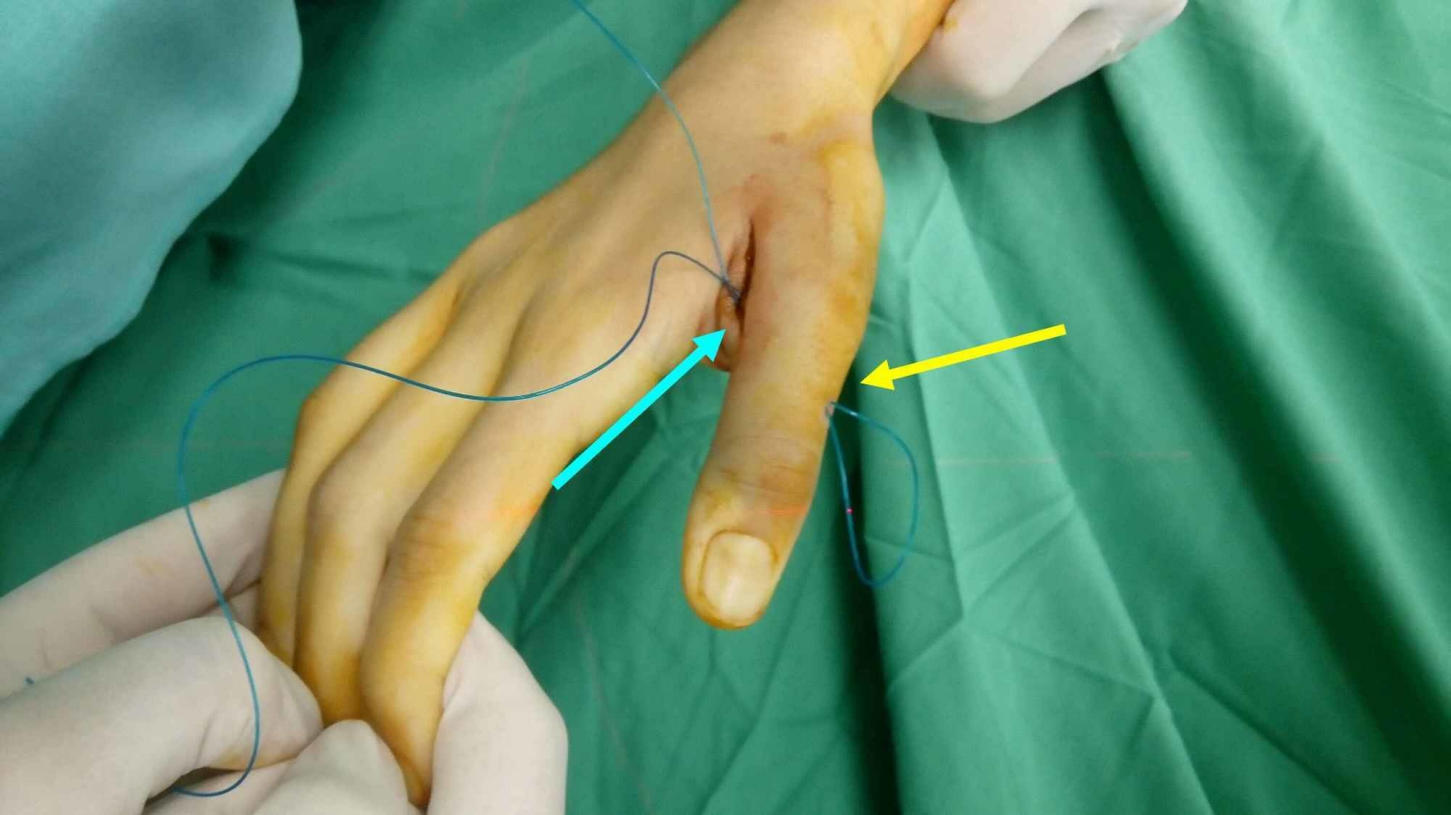
Intraoperative view of the transosseous suture The yellow arrow indicates the loop of the suture, which will be in contact with the lateral cortex. The blue arrow indicates the free ends of the transosseous suture after removing the transosseous needles.

**Figure 5:**
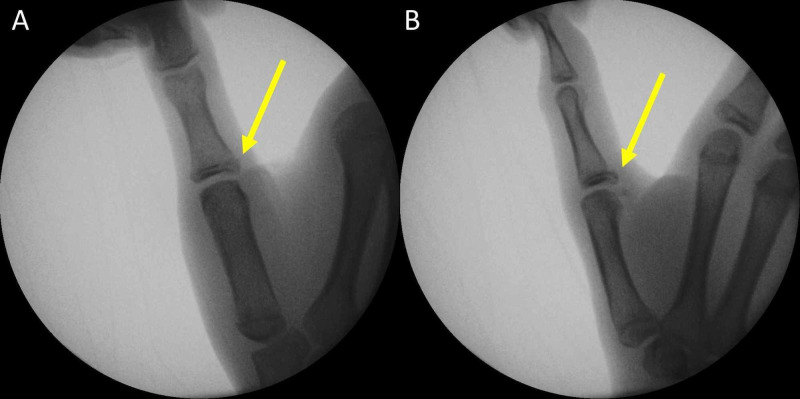
X-rays of the thumb after completion of surgery Anatomic reduction of the avulsed attachment of the UCL is seen on the anteroposterior (A) and lateral (B) x-ray views of the operated thumb. The arrows indicate the location of the fracture. UCL: ulnar collateral ligament

One non-absorbable, size 1, nylon suture was passed from proximal to distal through the one needle and returned through the other. The needles were removed. Blunt dissection with a small mosquito clamp from the radial skin incision to the radial cortex of the proximal phalanx was carried out, in order to allow direct contact of the suture loop with the radial cortex. The free ends of the suture were pulled and tied. Internal fixation of the bony avulsion of the UCL with the base of the proximal phalanx of the thumb by means of transosseous sutures was completed (Figures 4, 5).

The TMP joint was stable on valgus in extension and in 30 degrees of flexion. The wound was closed with 3.0 nylon mattress sutures. A short-arm splint with a thumb spica was placed, protecting the thumb from abduction and valgus. Stitches were removed after two weeks. The splint was removed after one month. A removable splint with a thumb spica was placed, and the patient started physical therapy. Rehabilitation was uneventful. Unrestricted return to previous activities was accomplished at three months postoperatively.

## Discussion

The aim of any surgical treatment of unstable complete ruptures of the UCL is to restore stability of the TMP joint, and hence to restore pinch strength, grip strength, and hand function [3]. Furthermore, if the bony avulsion contains a significant portion of the joint surface, anatomic restoration and not excision of the bony fragment is the preferred treatment [4]. Preoperative assessment leads to the selection of the appropriate surgical technique [7].

Various surgical techniques are available, such as direct primary repair, suture anchors, interference screws, transosseous sutures with or without a pull-out technique, arthroscopic-assisted reduction of the avulsed fragment, and reconstruction using autografts or allografts. In case of chronic degenerative arthritis, fusion of the TMP joint is the only option to deal with chronic pain and hand malfunction [3,7].

A pull-out technique using a button in direct contact with the skin on the radial side of the thumb was widely used in the past for the treatment of acute injuries of UCL [8]. However, this method may be accompanied by several complications, such as infection, cutaneous nerve compression, or progressive loosening of the fixation of UCL. These complications arise from the suture, which traverses the skin, and from direct pressure of the button on the skin [9].

In the presented case, the avulsed bony fragment comprised almost one-third of the joint surface. Thus, anatomic reduction and stable internal fixation was decided. Rein et al. described a similar technique, but the suture was passed only through the proximal phalanx of the thumb. In fact, it was used like a suture of a bony anchor to fix the avulsed stump of UCL to its anatomic attachment. Small size avulsion fractures were treated as non-osseous avulsions. For large bony avulsions, Rein et al. recommended additional fixation either with K-wires or with a small 1.1-1.3 mm screw, but such cases were not presented in their study [9].

In case of a large bony avulsion of the UCL, anatomic fixation of the fracture will automatically restore TMP joint stability. The presented surgical technique is an internal fixation technique of the bony avulsion of UCL using transosseous sutures.

## Conclusions

A simple surgical technique for the internal fixation of a bony avulsion of the distal attachment of the UCL of the TMP joint using transosseous sutures is presented. Early anatomic restoration of the bony avulsion leads to rapid healing of UCL, and hence to early restoration of normal function. Furthermore, the presented surgical technique avoids the complications of a pull-out button technique and the high cost of suture anchors.
